# Ergosterol and Its Metabolites Induce Ligninolytic Activity in the Lignin-Degrading Fungus *Phanerochaete sordida* YK-624

**DOI:** 10.3390/jof9090951

**Published:** 2023-09-20

**Authors:** Jianqiao Wang, Ru Yin, Yuki Hashizume, Yasushi Todoroki, Toshio Mori, Hirokazu Kawagishi, Hirofumi Hirai

**Affiliations:** 1 Key Laboratory for Water Quality and Conservation of the Pearl River Delta, Ministry of Education, School of Environmental Science and Engineering, Guangzhou University, Guangzhou 510006, China; wangjq@gzhu.edu.cn; 2 Graduate School of Science and Technology, Shizuoka University, 836 Ohya, Suruga-ku, Shizuoka 422-8529, Japan; yin.ru.21@shizuoka.ac.jp; 3 Faculty of Agriculture, Shizuoka University, 836 Ohya, Suruga-ku, Shizuoka 422-8529, Japan; hashi0187255@gmail.com (Y.H.); todoroki.yasushi@shizuoka.ac.jp (Y.T.); mori.toshio@shizuoka.ac.jp (T.M.); kawagishi.hirokazu@shizuoka.ac.jp (H.K.); 4 Research Institute of Green Science and Technology, Shizuoka University, 836 Ohya, Suruga-ku, Shizuoka 422-8529, Japan; 5 Research Institute for Mushroom Science, Shizuoka University, 836 Ohya, Suruga-ku, Shizuoka 422-8529, Japan; 6 Faculty of Global Interdisciplinary Science and Innovation, Shizuoka University, 836 Ohya, Suruga-ku, Shizuoka 422-8529, Japan

**Keywords:** lignin degradation, white-rot fungi, *Phanerochaete sordida* YK-624, inducing compounds

## Abstract

White-rot fungi are the most important group of lignin biodegraders. *Phanerochaete sordida* YK-624 has higher ligninolytic activity than that of model white-rot fungi. However, the underlying mechanism responsible for lignin degradation by white-rot fungi remains unknown, and the induced compounds isolated from white-rot fungi for lignin degradation have never been studied. In the present study, we tried to screen ligninolytic-inducing compounds produced by *P. sordida* YK-624. After large-scale incubation of *P. sordida* YK-624, the culture and mycelium were separated by filtration. After the separation and purification, purified compounds were analyzed by high-resolution electrospray ionization mass spectrometry and nuclear magnetic resonance. The sterilized unbleached hardwood kraft pulp was used for the initial evaluation of ligninolytic activity. Ergosterol was isolated and identified and it induced the lignin-degrading activity of this fungus. Moreover, we investigated ergosterol metabolites from *P. sordida* YK-624, and the ergosterol metabolites ergosta-4,7,22-triene-3,6-dione and ergosta-4,6,8(14),22-tetraen-3-one were identified and then chemically synthesized. These compounds significantly improved the lignin-degrading activity of the fungus. This is the first report on the ligninolytic-inducing compounds produced by white-rot fungi.

## 1. Introduction

The main components of lignocellulosic biomass are cellulose, hemicellulose, and lignin [1]. Lignin, a complex aromatic heteropolymer, is a constituent of plant cell walls and serves numerous biological functions in plant growth and metabolic processes. Additionally, lignin acts as a natural physical barrier, playing a crucial role in both passive and active defense mechanisms. Consequently, polysaccharide-degrading enzymes, such as cellulase enzymes, encounter difficulties in accessing and binding to lignin, leading to their deactivation. For these reasons, practical application technology for lignin biodegradation has not yet been applied in industry despite the numerous research investigations in this area [2,3,4].

There are some biomass pretreatment methods for lignin elimination, such as steam explosion, or acid or alkali treatment [5]. However, biological treatment of lignin under normal temperature and pressure with a low environmental load is desired [6]. In nature, white-rot fungi are the most important group of lignin biodegrades and can oxidize and fully mineralize lignin [7]. Lignin biodegradation by white-rot fungi is considered to occur as follows: (1) lignin-degrading enzymes (e.g., lignin peroxidase (LiP), manganese peroxidase (MnP) and hydrogen peroxide-generating enzymes (e.g., glyoxylate oxidase) are secreted under fertile nutrient conditions for the oxidation of lignin in wood; (2) the small-molecule lignin fragments, such as vanillin, which are produced by lignin depolymerization, are taken into the cell and decomposed into water and carbon dioxide by aromatic ring metabolic enzyme systems [8,9]. Considerable research has been conducted to elucidate the metabolic pathways employed by white-rot fungi for lignin degradation. However, the biochemical reactions involved in the conversion of lignin into carbon dioxide have received relatively little attention. Until the publication by Cerro et al. in 2021, the exact mechanism underlying lignin degradation by white-rot fungi remained elusive. In their groundbreaking study, Cerro et al. conclusively demonstrated the capacity of white-rot fungi to modify and utilize aromatic lignin-deconstruction products as carbon sources using ^13^C-isotope labeling [10]. Despite this progress, there is still a substantial gap in our understanding of how to significantly enhance the ligninolytic activity of white-rot fungi or the lignin-degrading capabilities of other microorganisms. Moreover, the induced compounds for lignin degradation have never been studied.

*Phanerochaete sordida* YK-624 has higher ligninolytic activity than that of model white-rot fungus *P*. *chrysosporium* [11]. In the present study, we tried to screen the ligninolytic-inducing compounds produced by *P. sordida* YK-624. After separation and purification, we identified the structures of compounds and determined their lignin-degrading activity. Investigations on the white-rot fungi’s metabolites affiliated with the ligninolytic system are highly limited [10,12]. Thus, this is the first report on ligninolytic-inducing compounds produced by white-rot fungi.

## 2. Materials and Methods

### 2.1. Fungus Cultivation

*P. sordida* YK-624 (ATCC 90872) was used. After 3 days of preincubation on potato dextrose agar (PDA) plates at 30 °C, the growing edge of the 10 mm diameter mycelium disks of *P. sordida* YK-624 was punched out. Ten disks were inoculated into 50 mL of high nitrogen and carbon (HN-HC) Kirk medium and then incubated for 3 weeks at 30 °C [13].

### 2.2. Screening of Ligninolytic-Inducing Compounds

After large-scale (5 L) incubation of *P. sordida* YK-624, the culture and mycelium were separated by filtration. The culture was concentrated under reduced pressure and then partitioned first with hexane and H_2_O, second with ethyl acetate (EtOAc) and H_2_O, and third with 1-butanol and H_2_O. The mycelium was freeze-dried and partitioned with hexane, EtOAc, methanol (MeOH) and H_2_O by solid–liquid extraction.

The hexane-, EtOAc- and MeOH-soluble constituents of the mycelia were applied to test their improvement of lignin-degrading activity and then used for further experiments. The hexane-soluble extract (129.5 mg) was fractionated by preparative thin-layer chromatography (TLC, DC-Alufolien Kieselgel 60F_254_, Merck, Darmstadt, Germany) to obtain 12 fractions. Fraction 6 (19.2 mg) was further separated by Sep-pak (Silica cartridges, Waters, MA, USA) with CH_2_Cl_2_ and MeOH to give compound **1**. 

The purified compound was analyzed by high-resolution electrospray ionization mass spectrometry (HR-ESI-MS, JMS-T100LC mass spectrometer, Thermo Fisher Scientific, San Jose, CA, USA) and nuclear magnetic resonance (NMR), which was conducted using a Jeol Lambda-500 spectrometer (JEOL, Tokyo, Japan) at 500 MHz and 125 MHz for ^1^H-NMR and ^13^C-NMR spectral analysis, respectively.

### 2.3. Identification of Ergosterol Metabolites

Low nitrogen and high carbon (LN-HC) Kirk medium was used for ergosterol metabolite determination [13]. The shaking cultivation of *P. sordida* YK-624 was conducted as described by Kondo et al. [14]. After 5 days of preincubation, ergosterol (finial concentration: 0.5 mM) was added and further incubated for 10 days. The mycelium was freeze-dried and partitioned with hexane by solid–liquid extraction, as described for the detection of ergosterol. The hexane-soluble extract (1.13 g) was fractionated by silica gel flash column chromatography (hexane/EtOAc/MeOH = 10/0/0; 9/1/0; 8/2/0; 7/3/0; 6/4/0; 5/5/0; 4/6/0; 3/7/0; 2/8/0; 1/9/0; 0/10/0; and 0/0/10, 0.5 L each) to give 10 fractions (fractions 10-1 to 10-10). Fraction 10-4 (434.2 mg) was separated by Sep-pak (Waters, ODS cartridges, 90% MeOH, MeOH and CH_2_Cl_2_) to obtain 3 fractions (fractions 10-4-1 to 10-4-3). Fraction 10-4-2 (197.3 mg) was further separated by NP-HPLC (COSMOSIL 5SL-II, *ϕ* 20 mm × 250 mm, hexane/CHCl_3_ = 8/2) to obtain two ergosterol metabolites, compound **2** and compound **3**. 

### 2.4. Determination of Ligninolytic Properties

The sterilized unbleached hardwood kraft pulp (UKP; *ϕ* 15 mm) was used for the initial evaluation of ligninolytic activity. Each soluble part was diluted to 0.1 mg μL^−1^ in acetone and 50 µL of each soluble part was used to saturate the UKP. Each compound (**ergosterol**, compound **2** and compound **3**) was prepared to 0.125, 0.5, 1.0, 1.5, 2.0, and 2.5 μmol in acetone. After solvent evaporation, the UKP was placed on a 12-well cell culture plate containing 3 mL of medium (1.5% agar, pH 5.0). A 5 mm diameter mycelium disk of *P. sordida* YK-624 was inoculated into the 12-well cell culture plate and then incubated at 30 °C. The lignin-degrading activity was evaluated by observing the effect of UKP bleaching.

The ligninolytic property under the presence of these compounds was further determined in detail. Wood media containing 0.5 g extractive-free beech wood meal (80~100 mesh) and 1.25 mL distilled water were sterilized, and then 1 mL of each compound was added at concentrations of 0.001, 0.01, 0.1, and 1 mM (acetone) and set in a sterile environment until the acetone was completely evaporated. A 10 mm diameter mycelium disk of *P. sordida* YK-624 was punched out and inoculated into the wood medium. The weight loss and lignin content of the wood meal samples were determined after two weeks of incubation using our previous method [11]. 

### 2.5. Synthesis of Ergosterol Metabolites 

**Figure 1:**
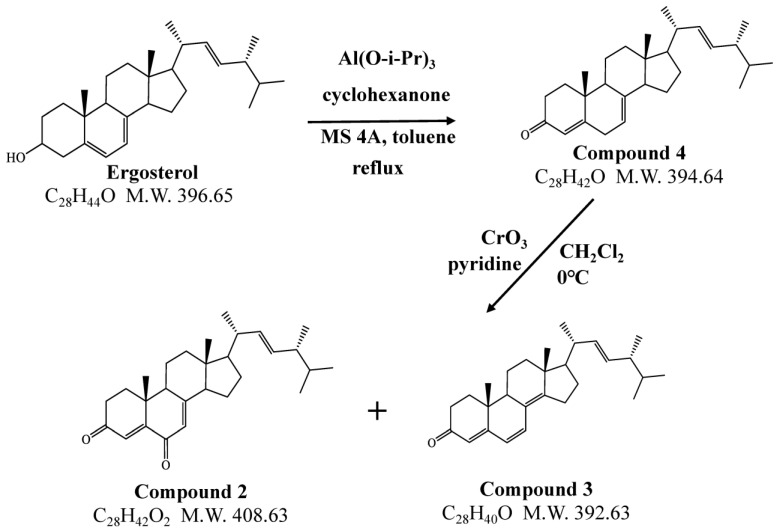
Synthesis of ergosterol metabolites. Compound **4**: ergosta-4,7,22-trien-3-one; compound **2**: ergosta-4,7,22-triene-3,6-dione; compound **3**: ergosta-4,6,8(14),22-tetraen-3-one.

The ergosterol metabolites were synthetized according to Kovganko and Sokolov [15]. Briefly, a mixture of ergosterol (5 g), cyclohexanone (18.3 mL), and 4 Å molecular sieves (5 g) in 350 mL toluene was boiled with stirring. The samples were then boiled for another 30 min after adding aluminum isopropoxide (13.2 g) and 5 mL toluene. H_2_O and a 2 M caustic soda solution were added after cooling to room temperature and then filtered. The filtrate was evaporated and then subjected to silica gel chromatography, with elution by hexane and EtOAc (10:1). Compound **4** (3.12 g) was obtained (Figure 1). The yield was 63%. The ^1^H NMR spectrum is shown in Figure S1. Compound **4** was used for further synthesis. Chromium trioxide (6 g) was added slowly to a mixture of 40 mL pyridine and 200 mL CH_2_Cl_2_ and stirred for 15 min. Compound **4** (3 g), which was dissolved in CH_2_Cl_2_ (67 mL), was added. The mixture was stirred at 0 °C for 2 h and then filtered. The filtrate was evaporated and then subjected to silica gel chromatography with elution by hexane and EtOAc (from 20:1 to 8:1). This gave 1.17 g of compound **2** and 0.4 g of compound **3**. The ^1^H NMR spectra are shown in Figures S2 and S3. 

## 3. Results

In this study, we tried to screen the ligninolytic-inducing compounds from *P. sordida* YK-624. The culture was partitioned with hexane, EtOAc and 1-butanol, and the mycelia was partitioned with hexane, EtOAc and MeOH. These soluble fractions were used for further assessment in this study. The results of the initial evaluation of ligninolytic activity suggested that the hexane-, EtOAc- and MeOH-soluble extracts from the mycelium had an effect on UKP bleaching.

We detected compound **1** from the hexane-soluble extract. The structure was identified by ESI-TOF-MS and NMR analyses. Based on HR-ESI-MS, the molecular formula of compound **1** was C_28_H_44_O (*m/z* 377.06113 [M-H-H_2_O]^−^). Together with the ^1^H-NMR spectrum, compound **1** was determined to be ergosterol (Figure S4).

The results of the initial evaluation of ligninolytic activity suggested that ergosterol accelerated UKP bleaching. Ergosterol is a component of the fungal cell membrane; thus, we considered that the metabolites of ergosterol might promote lignin degradation. We further investigated ergosterol metabolites from *P. sordida* YK-624 with the addition of ergosterol, and new peaks were observed by HPLC. A metabolite of ergosterol was isolated from the hexane-soluble extract of the mycelia. Based on the ESI-TOF-MS and NMR analyses, the metabolite was determined to be ergosta-4,6,8(14),22-tetraen-3-one (compound **3**, Figure 1).

**Figure 2:**
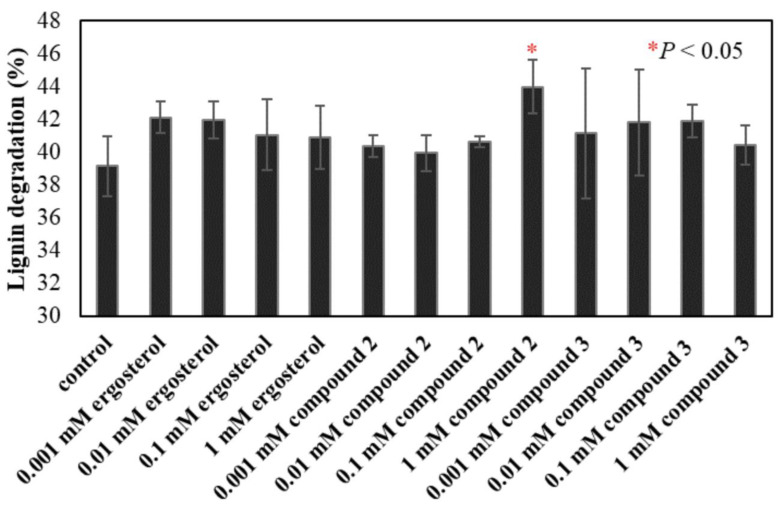
Effect of ergosterol and its metabolites on lignin degradation by *P. sordida* YK-624. Values are means ± SD of triplicate samples.

However, the yield of compound **3** was not sufficient for determining its ligninolytic-inducing activity; thus, we synthesized compound **3**. During the synthesis, another compound, ergosta-4,7,22-triene-3,6-dione (compound **2**, Figure 1), which was similar to ergosterol, was obtained. By comparing the HPLC and MS results of the hexane-soluble extract, it was identified as a plausible ergosterol metabolite. The lignin-degrading activity of *P. sordida* YK-624 with the addition of ergosterol and its metabolites was investigated. The ligninolytic activity significantly increased with the addition of ergosterol and its metabolites (Figure 2). These results indicated that ergosterol and its metabolites could improve lignin-degrading activity.

## 4. Discussion

In response to global climate change and the need for sustainable development, it is imperative to explore renewable energy sources and establish a viable biobased economy [16]. Lignin, a complex organic polymer, is the second most abundant biomass resource on Earth, surpassed only by cellulose [17]. Lignin plays a crucial role in providing mechanical support and enhancing the rigidity of wood, making it resistant to decay. However, the degradation and mineralization of lignin are primarily carried out by white-rot fungi, making them the only organisms capable of effectively breaking down this substantial natural carbon and energy reservoir [18]. Therefore, it is essential to enhance the lignin-degrading capacity of white-rot fungi to optimize the utilization of lignocellulosic biomass resources. Consequently, there has been a growing focus on investigating lignin biosynthesis and its modification mechanisms for various industrial applications, including improving forage digestibility, increasing biofuel productivity, and enhancing bioproduct synthesis efficiency, over the past decade [19,20,21].

In this study, we successfully isolated and identified ligninolytic-inducing compounds from *P. sordida* YK-624. Among the isolated compounds, ergosterol stands out as a significant component of fungal cell membranes, playing an important role in maintaining membrane integrity, fluidity, fluidity, and permeability [22]. Numerous studies have highlighted the importance of ergosterol in fungal cell growth, proliferation, environmental stress response, cellular detoxification, nutrient transport, and host–pathogen interactions [23]. It has been reported that ergosterol can enhance stress resistance in *Saccharomyces cerevisiae* when exposed to D-limonene [24]. Additionally, ergosterol has been suggested as a key compound responsible for the anti-neuroinflammatory effects of *Auricularia polytrichas* against bisphenol A induction [25]. Moreover, ergosterol has gained considerable attention in pharmacological research due to its potential health benefits, including its impact on oxidation, immune function, diabetes, cancer, and other diseases [26]. Notably, ergosterol has been identified as the primary molecule inducing cancer cell death in *Amauroderma rude* and has been shown to induce apoptosis in breast cancer cells by inhibiting their vitality through activation of the apoptotic signaling pathway [27].

In biotechnological research, ergosterol is commonly utilized as a growth biomarker to quantify fungal biomass in solid substrates, exhibiting a positive correlation [28]. Interestingly, ergosterol has been identified as an indicator for evaluating the growth of *Lentinus crinitus* during soil bioremediation processes. Notably, the ergosterol rate of this fungus remains unaffected by the culture conditions [29]. In this study, ergosterol was identified as one of the inducers of lignin-degrading activity in *P. sordida* YK-624. A previous investigation demonstrated that *Inonotus obliquus* efficiently colonized wheat straw, exhibiting a high ergosterol content of 280 μg/g dry weight and achieving 45% lignin degradation after a 15-day fermentation period. Additionally, a significant concentration of lignin-degrading enzymes, including Lip, MnP, and laccase, was observed [30]. These findings further support a positive correlation between ergosterol and lignin degradation capacity during the lignin degradation process.

Additionally, we investigated the ergosterol metabolites from *P. sordida* YK-624 and successfully identified two compounds: ergosta-4,7,22-triene-3,6-dione and ergosta-4,6,8(14),22-tetraen-3-one. Ergosta-4,7,22-triene-3,6-dione has received limited research attention and was originally isolated from *Ganoderma lucidum* by Hirotani et al. [31]. The compound **3** (ergosta-4,6,8(14), 22-tetraen-3-one) we found has been previously isolated and identified as a metabolite of ergosterol in the mushroom *Polyporus umbellatus* [32]. In a previous study, ergosta-4,6,8(14), 22-tetraen-3-one was shown to have immunomodulatory properties and an inhibitory effect on tumor and cancer cells [33]. It was also identified in an Antarctic fungus *Cortinarius xiphidipus*, and its cytotoxic effect on four tumor cells was evaluated [34]. Ergosta-4,6,8(14),22-tetraen-3-one demonstrated the ability to prevent early renal injury in rats with aristolochic acid I-induced nephropathy, resulting in fewer lesions [35]. Additionally, this compound was obtained from the medicinal fungus *Pholiota adiposa* and showed potential as an effective ingredient for preventing and treating diabetes [36]. Until now, research on ergosta-4,6,8(14),22-tetraen-3-one has been primarily focused on pharmacological studies. Interestingly, both metabolites significantly enhance the lignin-degrading activity of the fungus, marking the first report on ligninolytic-inducing compounds produced by white-rot fungi.

## 5. Conclusions

In conclusion, we isolated and identified the ligninolytic-inducing compounds from *P. sordida* YK-624. Furthermore, these three compounds have been proven to have a positive impact on the lignin-degradation of *P. sordida* YK-624. The findings obtained from this study could potentially contribute to the advancement of a comprehensive bioprocess for lignin in various industrial applications.

## Data Availability

Not applicable.

## Supplementary Materials

The following supporting information can be downloaded at: https://www.mdpi.com/article/10.3390/jof9090951/s1, Figure S1: ^1^H NMR spectrum of the compound **4** (CDCl_3_); Figure S2: ^1^H NMR spectrum of the compound **2** (CDCl_3_); Figure S3: ^1^H NMR spectrum of the compound **3** (CDCl_3_); Figure S4: ^1^H NMR spectrum of the compound **1** (CDCl_3_).
