# Supplementary material for: Ergosterol and Its Metabolites Induce Ligninolytic Activity in the Lignin-Degrading Fungus Phanerochaete sordida YK-624
Source: J Fungi (Basel). 2023 Sep 20;9(9):951. doi: 10.3390/jof9090951 (PMC10532932; doi:10.3390/jof9090951)
Supplement: Supplementary file 1 [file jof-09-00951-s001.zip › jof-2608183-supplementary.pdf]

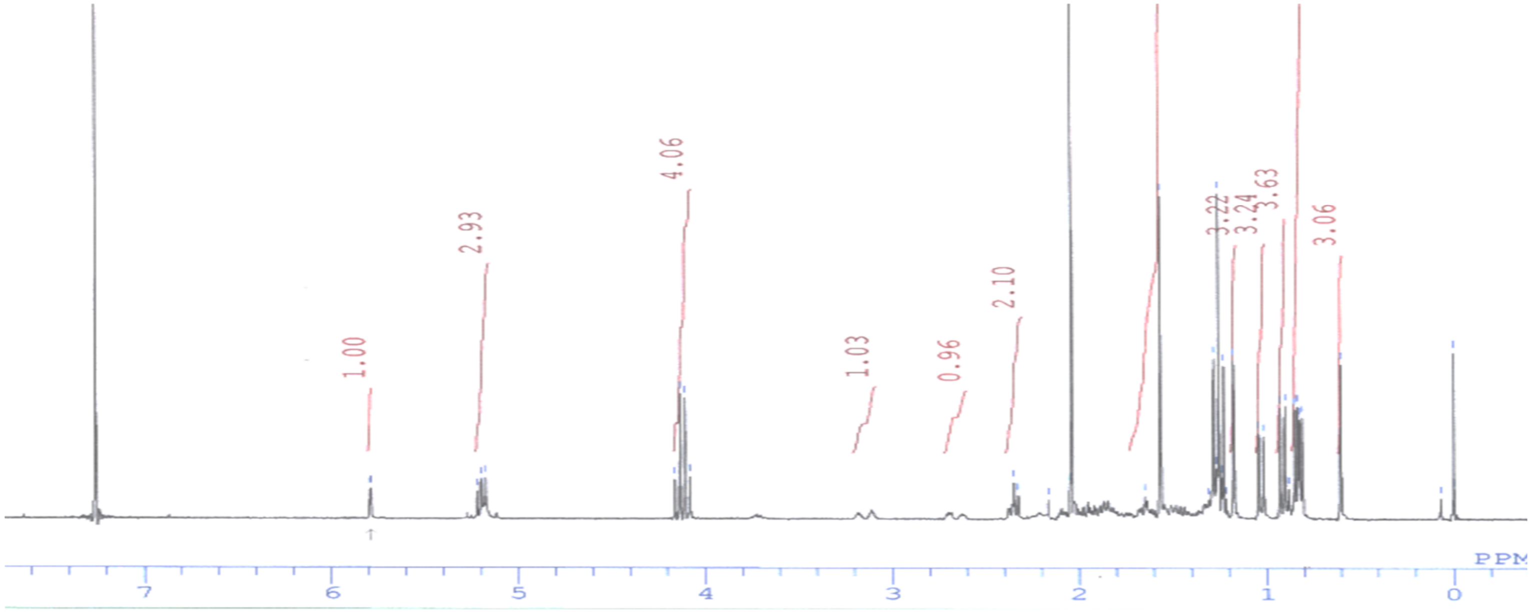

Figure S1. ^{1}H NMR spectrum of the compound 4 ($CDCl_3$).

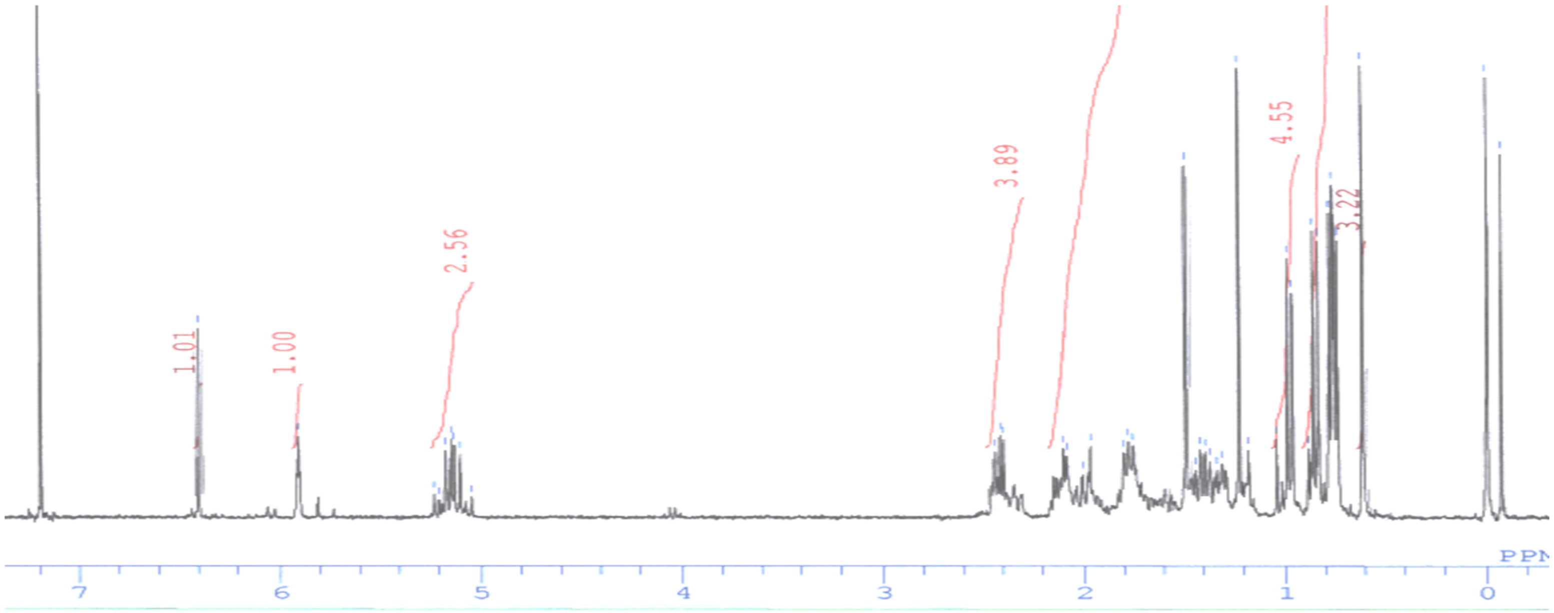

Figure S2. ^{1}H NMR spectrum of the compound 2 ($CDCl_3$).

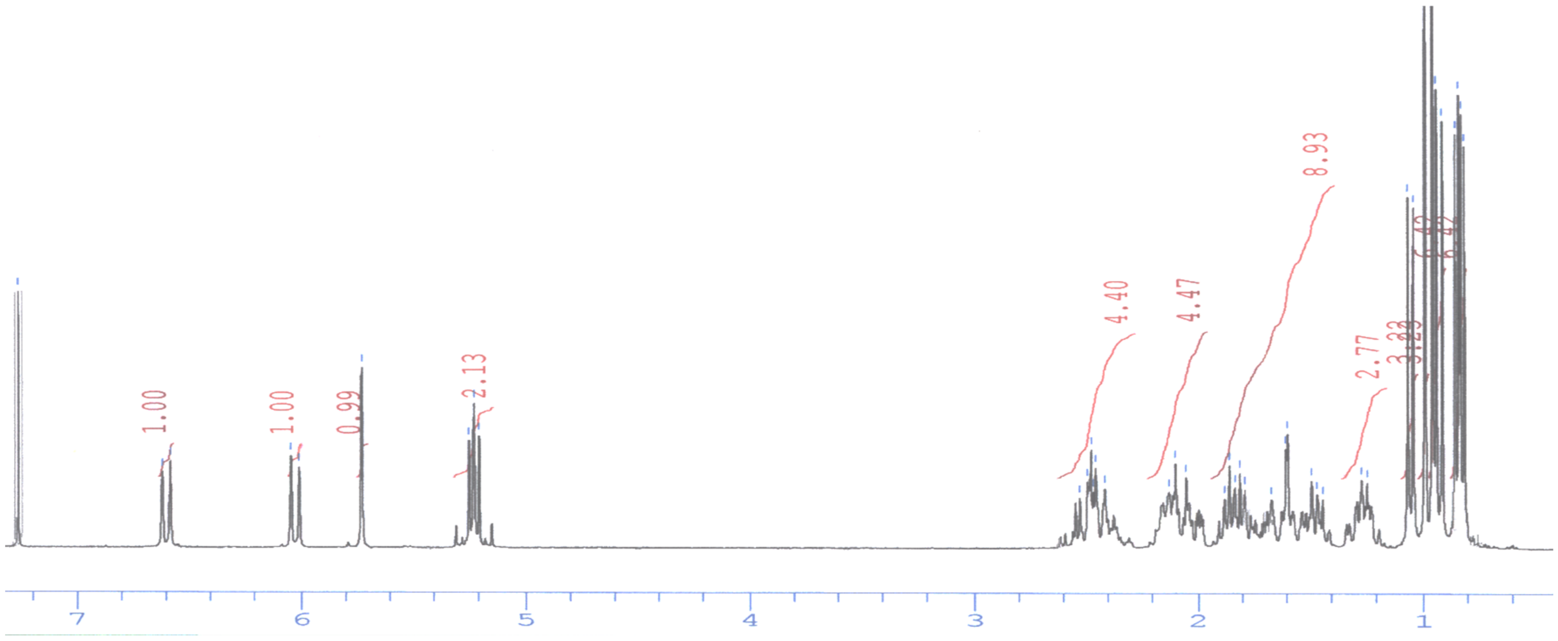

Figure S3. ^{1}H NMR spectrum of the compound 3 ($CDCl_3$).

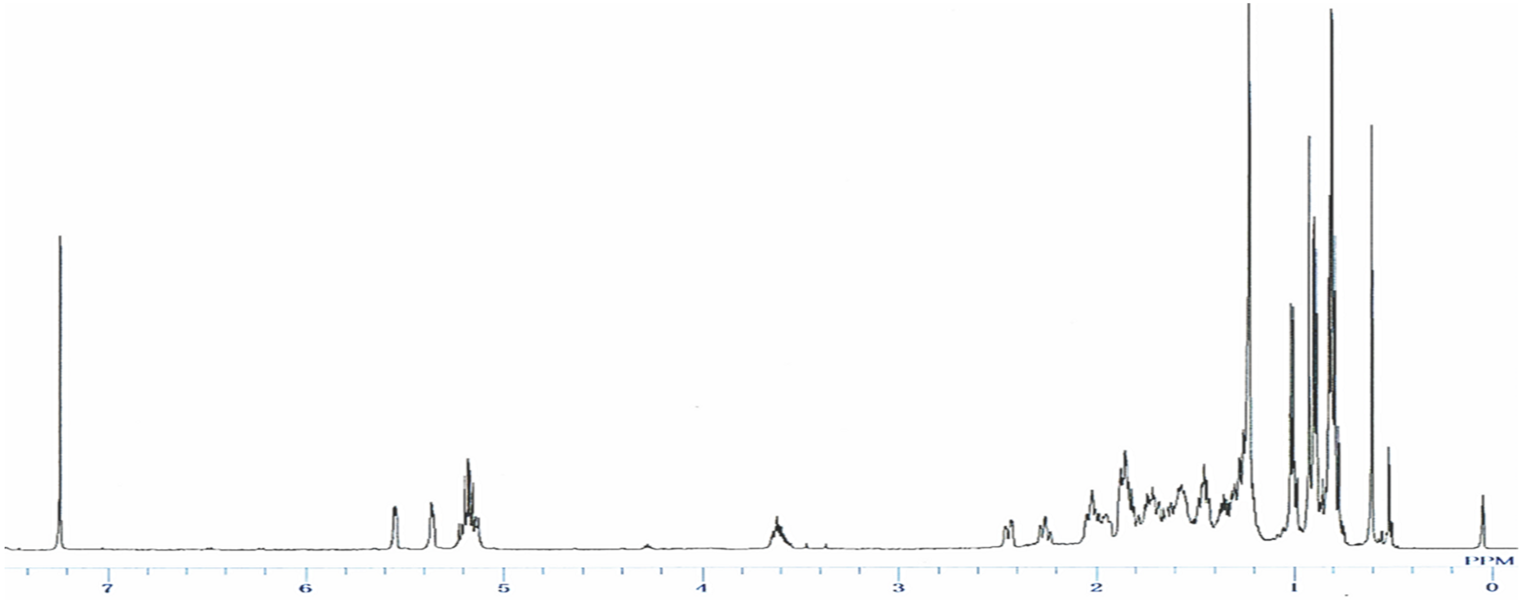

Figure S4. ^{1}H NMR spectrum of the compound 1 ($CDCl_3$).
